# Utilizing chia flour‐based gelled emulsion for the development of functional beef patties with enhanced nutritional profile

**DOI:** 10.1002/fsn3.4350

**Published:** 2024-07-19

**Authors:** Meltem Serdaroğlu, Özlem Yüncü‐Boyacı, Hülya Serpil Kavuşan

**Affiliations:** ^1^ Food Engineering Department, Engineering Faculty Ege University Bornova Izmir Turkey

**Keywords:** beef patty, chia flour, fat replacer, gelled emulsion, peanut oil

## Abstract

This study evaluated chia flour, egg white powder, and peanut oil gelled emulsion (GE) as a fat replacer in beef patties. Four formulations were prepared, replacing beef fat with different levels of gelled emulsion: 0% (C), 50% (G50), 75% (G75), and 100% (G100). The beef patties with GE showed improved nutritional properties, technological parameters, and cooking characteristics. A remarkable reduction in SFAs was achieved with the substitution of beef fat by the GE, with reductions of 33.71%, 46.64%, and 72.04% for 50%, 75%, and 100% substitution levels, respectively. AI and TI indices decreased, indicating healthier profiles. Reformulated samples exhibited lower hardness, gumminess, and chewiness values. Color and appearance were similar to the control, with higher sensory scores for G75 and G100. GE impacted color parameters, increasing L* and b* values. The utilization of GE effectively minimized voids in the beef patty structure, leading to improved cooking yield and a more compact structure. GE influenced oxidative stability, with average onset temperatures of 126.33°C (50% GE), 140.58°C (75% GE), and 127.04°C (100% GE). In conclusion, gelled emulsions could successfully contribute to producing healthier meat products.

## Graphical abstract

This study investigated the use of chia flour, egg white powder, and peanut oil gelled emulsion (GE) as a fat substitute in beef patties, with formulations replacing beef fat at different levels (0%, 50%, 75%, and 100%). The incorporation of gelled emulsion resulted in improved nutritional, technological, and cooking properties of the beef patties. Notably, the substitution of beef fat with GE led to significant reductions in saturated fatty acids (SFAs) and improved indices related to health, while also enhancing sensory attributes and structural characteristics, demonstrating the potential of gelled emulsions in producing healthier meat products.

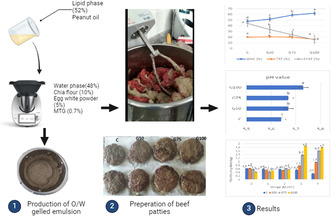

## 1 INTRODUCTION

Meat's nutritional significance arises from its provision of vital nutrients like amino acids, vitamins, and minerals (Pereira & Vicente, 2013). Nonetheless, concerns have emerged about its high fat content, saturated fatty acids (SFAs), and cholesterol levels, linked to increased risks of cardiovascular diseases and diabetes (WHO, 2009). Consequently, consumer awareness has driven the demand for low‐fat meat products with improved fatty acid composition (Serdaroğlu et al., 2016). Nevertheless, the decrease in fat and the integration of substitutes or vegetable oils have implications for technological and sensory properties, including factors like cooking loss, emulsion stability, and texture, ultimately influencing consumer acceptance (dos Santos Alves et al., 2016). In response to this, suggestions include the incorporation of dietary fibers, hydrocolloids, or polyunsaturated fatty acid‐rich oils to reduce the fat content and enhance the overall fatty acid profile (Jiménez‐Colmenero, 2007). Nevertheless, using liquid oils negatively impacts sensory and technological attributes (Serdaroğlu et al., 2016). Recent research suggests that gelled emulsions, hydrogels, and organogels offer potential solutions to these challenges (Lucas‐Gonzalez et al., 2023; Serdaroğlu, 2021).

Gelled emulsions are unique in their semi‐solid texture and are composed of a polymeric network consisting of proteins and/or polysaccharides. This network structure enables the dispersion of oil droplets, mimicking the textural properties typically provided by saturated fats in food products (Lucas‐Gonzalez et al., 2023). This approach offers an opportunity to improve the overall lipid profile by incorporating oils that are high in polyunsaturated fatty acids (PUFAs), thus providing a healthier alternative (de Souza Paglarini et al., 2019; Kavuşan et al., 2020; Öztürk‐Kerimoğlu et al., 2021; Serdaroğlu et al., 2017). However, limited research has been undertaken on the utilization of gelled emulsions containing chia flour as a replacement for animal fat in different meat formulations (de Souza Paglarini et al., 2019; Pintado et al., 2016). Additionally, a recent study has explored the production of GE formulated with chia flour and peanut oil (Badar et al., 2023).

Chia (*Salvia hispanica* L.) is a plant belonging to the *Lamiaceae* family, highly esteemed as a food source. (Sandoval‐Oliveros & Paredes‐López, 2013). Chia seeds are known for being rich in α‐linolenic acid (ALA), which has a beneficial ratio of omega‐3 to omega‐6. Their oil content is around 30%, and their protein content varies between 19% and 27%, featuring a favorable blend of essential amino acids. The seeds serve as a notable source of dietary fiber, ranging from 34% to 50%, surpassing the levels found in flaxseeds. Additionally, chia seeds encompass antioxidant compounds, including derivatives of caffeic acid such as rosmarinic acid, danshensu, and its glycosides, along with flavonoids like quercetin and kaempferol. The investigation of antioxidant activity is broad and encompasses its protective effects against oxidative damage in cells and tissues. Its potential role in preventing various diseases linked to oxidative stress, including cancer, diabetes, and cardiovascular problems, has been extensively studied (Sandoval‐Oliveros & Paredes‐López, 2013).

Peanut oil, derived from peanuts (*Arachis hypogaea*), is a nutritionally valuable oil with a favorable lipid composition, containing higher levels of unsaturated fatty acids (Nacak et al., 2021) compared to saturated fats, and it is free from trans fats and cholesterol (Akhtar et al., 2014). It also contains antioxidants, vitamin E, phytosterols, squalene, and p‐coumaric acid, which contribute to its health‐promoting properties. While the composition and health effects of peanut oil as a food ingredient have been extensively studied, there is limited research on its utilization as an animal fat replacer in meat products (Marquez et al., 1989; Nacak et al., 2021; Öztürk‐Kerimoğlu et al., 2021; Shao et al., 2023; Wongpattananukul et al., 2022). The existing gap in the literature has spurred the present study, which seeks to explore the effects of substituting beef fat with a gelled emulsion containing chia flour and peanut oil on the physicochemical, technological, textural, oxidative, and sensory characteristics of beef patties.

## 2 MATERIALS AND METHODS

### 2.1 Materials

Post‐rigor beef samples from the Holstein breed (male, 19 months old, carcass weight 310 kg) were obtained in the form of boneless rounds. The analysis of triplicate samples showed the following composition: 72.6% moisture, 21.1% protein, 4.8% fat, and 1.5% ash. These beef samples, along with beef fat (31% palmitic, 19% stearic, 46% oleic, 3% linoleic, and 1% other) were generously provided by MIGROS TRADE INC. Peanut oil, which consisted of 44.8% oleic acid, 32% linoleic acid, 9.5% palmitic acid, 3.6% stearic acid, 2.5% behenic acid, 1.9% arachidic acid, and 5.7% other fatty acids, was acquired from Smart Kimya Ltd. Şti. (Izmir, Turkey). The defatted chia flour (*Salvia hispanica* L.), sourced from Tazemiz company in Mersin, Turkey, is rich in various bioactive compounds, including gancaonin V, 8‐prenyllepidissipyrone, 4‐hydroxycoumarin, butyl 3‐O‐caffeoylquinate, 9,10‐dihydro‐10‐(3,4‐dihydroxy phenyl)‐pyrano[2,3‐h]catechin‐8‐one, caffeic acid, ferulic acid, kaempferol 3‐xylosyl‐(1→2)‐rhamnoside, kaempferol 3‐p‐coumarate, 5‐hydroxy‐7,2′,3′,4′,5′‐pentamethoxyflavone, and salvianolic acid L. The salt and spice mixes were sourced from the local market in İzmir, while the egg white powder was obtained from Dr. Gusto Ltd. Şti. (Istanbul, Turkey). The emulsifying agents used in the gel emulsion formulation, namely PGPR (polyglycerol polyricinoleate) and MTG (microbial transglutaminase), were supplied by Çağdaş Kimya A.Ş. (Istanbul, Turkey). The experimental work was conducted at the pilot plant and laboratories of the Food Engineering Department at Ege University (Izmir, Turkey). All other reagents employed in this study were of analytical grade and were sourced from Sigma Aldrich Co. Ltd. (St. Louis, MO, USA) and Merck (Germany).

### 2.2 Production of O/W gelled emulsion

The O/W (oil‐in‐water) gelled emulsion was produced with the cold gelation method, following the procedure outlined by Pintado et al. (2015). For the water phase (W), a blend of chia flour (10%), egg white powder (EWP) (5%), and water was homogenized using a Thermomix (Vorwerk, Germany) at a speed of 5600 rpm for 45 s. Subsequently, 0.7% MTG was incorporated into the mixture, and homogenization continued for an additional 15 s. The oil phase (O) based on peanut oil was introduced to the water phase, and emulsification was achieved using a high‐speed mixer (Thermomix) at 5600 rpm for 3 min. The resulting emulsions were then incubated at a temperature of +4°C for 12 h.

### 2.3 Experimental design and preparation of beef patties

Slices of boneless beef from the top round of cattle carcasses were passed through a 3 mm grinder (Arnica Promeat Grande, Turkey) plate to obtain ground beef. The ground beef was then mixed with fat and/or fat substitutes in the form of gelled emulsions, along with other additives, including 2% salt and 2% spice mixture, until a homogeneous mixture was achieved. The control patties were prepared with 100% beef fat, whereas for the modified‐fat patties, 50% (G50), 75% (G75), and 100% (G100) of the beef fat were replaced with GE. The mixing process was performed using a mixer (HYM360, Mateka, Turkey). Subsequently, the mixture was formed into patties with a metal mold with a thickness of 1.5 cm and a diameter of 100 mm. The formulation of the patty samples is provided in Table 1. A total of 3500 g of patty dough was prepared for each batch, and approximately 35 patties were produced per group and per replication. The process of producing beef patties was duplicated twice, resulting in two independent batches made on different days. The patty samples were stored in sealed polyethylene bags (without air space) at −18°C for 4 months. TBARS and color analyses were conducted at monthly intervals (0, 1, 2, 3, and 4 months) during a 4‐month storage period. All analyses, except for texture and sensory evaluations, were conducted on raw samples.

**TABLE 1:** Formulation of beef patties.

Treatments	Beef (%)	Beef fat (%)	O/W gelled emulsion (%)	Salt (%)	Spice mix^a^ (%)	Total (%)
C	76	20	–	2	2	100
G50	76	10	10	2	2	100
G75	76	5	15	2	2	100
G100	76	–	20	2	2	100

*Note*: C: patties prepared with 100% beef fat; G50: patties prepared with 50% gelled emulsion; G75: patties prepared with 75% gelled emulsion; G100: patties prepared with 100% gelled emulsion.

^a^ Spice mix: onion powder (2%), black pepper (1%) and cumin (1%).

### 2.4 Methods

#### 2.4.1 Analysis of the gelled emulsion

The gelled emulsion underwent centrifugation at 200 g for 3 min, followed by storage at +4°C, and the subsequent observation of the separation of the serum layer. To determine the stability against heat treatment, the emulsion was kept in a water bath at 70°C for 30 min, and phase separation was observed (Surh et al., 2007). A light microscope (Olympus CX21, Tokyo, Japan) with a 10× objective was utilized for the microscopic analysis of the O/W gelled emulsion.

#### 2.4.2 Proximate analysis and energy value

The moisture and ash contents of the samples were determined following the methods established by the AOAC in 2012. The fat content was determined through chloroform/methanol extraction, as specified by Flynn & Bramblett in 1975. The protein content was assessed utilizing the Dumas combustion method through the LECO (FP‐528, USA) protein/nitrogen analyzer. To determine the total energy value in kilocalories (kcal), Atwater values were applied, aligning with fat (9 kcal/g), protein (4.02 kcal/g), and carbohydrates (3.87 kcal/g), as specified by Mansour & Khalil in 2000.

#### 2.4.3 Batter stability

Water‐holding capacity (WHC) and emulsion stability (total expressible fluid (TEF) and expressible fat (EFAT)) were evaluated in triplicate using the methodology outlined by Hughes et al. (1997) with certain modifications.

#### 2.4.4 Cooking yield and cooking measurements

The percentage of cooking yield was assessed by calculating the weight difference between the patties before and after cooking (in a pre‐heated electric grill at 180°C, 3 min for each side). The cooking yield and fat retention were calculated based on the methodology established by Murphy et al. (1975). The reduction in patty diameter and thickness was measured using a Vernier caliper and calculated using the provided equations.

$$\begin{array}{c} \text{Reduction in patty diameter}\;\left(\%\right)=\\ \frac{\left(\text{Uncooked patty diameter}-\text{Cooked patty diameter}\right)}{\text{Uncooked patty diameter}}\times 100 \end{array}$$

$$\begin{array}{c} \text{Change in patty thickness}\;\left(\%\right)=\\ \frac{\left(\text{Uncooked patty thickness}-\text{Cooked patty thickness}\right)}{\text{Uncooked patty thickness}}\times 100 \end{array}$$

The cooking yield, moisture retention (El‐Magoli et al., 1996), and fat retention were calculated by weight differences for patties before and after cooking (Murphy et al., 1975) and calculated according to the following equations:

$$\begin{array}{c} \text{Moisture}\;\text{retention}\;\left(\%\right)=\\ \left(\%\;\text{Yield}\times \%\;\text{Moisture}\;\text{in}\;\text{cooked}\;\text{patty}\right)/100 \end{array}$$

$$\mathrm{Fat}\;\text{retention}\;\left(\%\right)=\frac{\left(\text{Cooked weight}\right)\times \left(\%\mathrm{Fat}\;\text{in cooked patty}\right)}{\;\left(\mathrm{Raw}\;\text{weight}\right)\times \left(\%\mathrm{Fat}\;\text{in}\;\mathrm{raw}\;\text{patty}\right)}\times 100.$$

Patty's dimensional shrinkage was calculated using the following equation.

$$\begin{array}{c} \text{Shrinkage}\;\left(\%\right)=[\left(\mathrm{Raw}\;\text{thickness}-\text{Cooked}\;\text{thickness}\right)+(\mathrm{Raw}\;\text{diameter}\\ \;-\text{Cooked}\;\text{diameter})/\left(\mathrm{Raw}\;\text{thickness}+\mathrm{Raw}\;\text{diameter}\right)]\times 100 \end{array}$$

#### 2.4.5 Fatty acid profile and cholesterol content

The lipid extraction from the patties was performed according to the established protocol as outlined by Flynn and Bramblett (1975). Subsequently, the extracted lipids underwent methylation using the precise method outlined by Paquot and Hautfenne (1987). Fatty acid methyl esters (FAME) analysis was conducted utilizing a gas chromatograph (GC 2010 Plus, Shimadzu Corp., Kyoto, Japan) fitted with a silica capillary column (SUPELCO SP TM‐2560) measuring 100 m in length, 0.25 mm in inner diameter, and with a 0.20‐μm film thickness. The helium injector and flame ionization detector (FID) were initially set at 140°C, followed by a temperature ramp from 140 to 250°C at a rate of 4°C/min. Subsequently, the temperature was maintained at 240°C for 10 min. The determination of the atherogenicity index (IA) and thrombogenicity index (IT) of the samples was carried out using the methodology defined by Ulbricht and Southgate (1991), employing the following formulations:

$$\mathrm{IA}=\left[C12:0+\left(4\times C14:0\right)+C16:0\right]/\sum \mathrm{UFA}$$

$$\begin{array}{c} \mathrm{IT}=\left(C14:0+C16:0+C18:0\right)/\\ \left[0.5\times \sum \text{MUFA}+\left(0.5\times \sum n-6\text{PUFA}\right)+\left(3\times \sum n-3\text{PUFA}\right)+\left(n-3/n-6\right)\right] \end{array}$$

The cholesterol content in the samples was assessed using a modified method derived from the procedures outlined by Yüncü et al. (2022). The calculation of the cholesterol content (mg/100 g) was performed utilizing the following equation:

$$\begin{array}{c} \text{Cholesterol content}\;\left(\mathrm{mg}/100\;g\right)=\\ \left[\left(0.711\times \left(A2-A1\right)/\text{sample weight}\;\left(g\right)\right)\times 100\times 25\right] \end{array}$$

#### 2.4.6 Instrumental parameters

The color profiles of beef patties were assessed using a colorimeter (CR‐200, Konica Minolta, Japan) that was calibrated using a black‐and‐white plate standard. For the measurements, the patties were sliced into 1 cm thick pieces and placed under illuminant D65 with a 10° observation angle. The color parameters, namely lightness (L*), redness (a*), and yellowness (b*), were recorded in triplicate from the cross‐sectional surface of the samples to ensure accuracy and consistency. Each sample surface was observed at an angle of 10° for 30 min (blooming time), and three measurements were recorded for each sample surface at each time interval. Texture profile analysis (TPA) was carried out to assess the texture characteristics of the samples. Using a texture analyzer (TA‐XT2, Stable Micro Systems, Haslemere, UK), each sample underwent four replications. Parameters including hardness (N), springiness, cohesiveness, gumminess (N), and chewiness (N·mm) were determined based on the force and time curves acquired during the analysis. The samples, measuring 1 cm × 1 cm × 1 cm in dimensions, were subjected to two compressions at 50% of their original height using a cylindrical aluminum probe with a diameter of 36 mm. The specific experimental conditions included a load cell of 5 kg, a post‐test speed of 2 mm/s, a cross‐head speed of 1 mm/s, and a test speed of 1 mm/s.

#### 2.4.7 Sensory assessment

The sensory panels were conducted in a dedicated sensory test laboratory that featured partitioned cabinets and adhered to controlled lighting conditions by ISO standards (ISO, 2007). Two separate sensory evaluation sessions (for two separate productions) were organized to evaluate the samples, and the panelists were asked to evaluate all samples. The same panelists were used in both sessions. Randomly selected samples from each formulation were assigned for sensory evaluation. Sensory assessment was conducted using a 9‐point hedonic scale, ranging from 9 (extremely liked) to 1 (extremely disliked). The panelists were instructed to evaluate the samples by considering various properties, including appearance, color, texture, juiciness, oiliness, flavor, and overall acceptance. Each assessment consisted of a panelist group comprising 25 individuals, with a gender distribution of 65% female and 35% male, aged between 21 and 34. Prior to the evaluation, each panelist was provided with a randomly coded beef patty sample, which was cooked (180°C, 8 min) in a pre‐heated electric grill (Premier, Turkey) before being served to the panelists.

#### 2.4.8 Oxidative stability

Over a 4‐month duration, patty samples were evaluated to ascertain their oxidative stability by tracking alterations in the concentrations of 2‐Thiobarbituric Acid Reactive Substances (TBARS). The TBARS value was quantified using the methodology established by Witte et al. (1970).

#### 2.4.9 Differential scanning calorimetry (DSC) measurements

The lipid phase was isolated from the samples using the method explained by Folch et al. (1957). To determine the onset temperature of oxidation using Differential Scanning Calorimetry (Q20, TA Instruments, USA), samples weighing 4.00 ± 0.01 mg were subjected to controlled heating with a linear temperature ramp of 10°C per minute within the temperature range of 20–250°C. The flow rate of oxygen was maintained at 50 mL/min, corresponding to the ambient pressure. The onset temperature of oxidation was ascertained by extending the baseline of the recorded temperature curve beyond the exothermic region associated with the oxidation reaction. This extension involved extrapolating the exothermic slope from the inflection point on the curve to an elongated baseline. The intersection point was then identified by utilizing TA Instruments Advantage Software (version 5.5), enabling the determination of the precise onset temperature of oxidation.

#### 2.4.10 Data analysis

The beef patty production procedure was replicated twice, resulting in two distinct batches produced on different days. We prepared four treatments (C, G50, G75, and G100) of beef patties in duplicate (*n* = 2) which were analyzed at five times periods (0, 1, 2, 3, and 4 months) during the storage at −18°C for TBARS, L*, a*, and b* values. Other analyses were conducted immediately after patty production. For each formulation, three patties per batch (approximately 35 patties of 100 g per repetition for each batch) were chosen for the subsequent analyses. Analysis of variance (ANOVA) using a general linear model (GLM) was employed, where the storage period and fat source were considered fixed effects, and replicate (production day) served as a random term. The same model was utilized for the analysis of sensory data, incorporating panelists as a random term. The obtained results were analyzed using the SPSS software package version 20.0 (SPSS Inc., Chicago, USA), and significant differences between means, along with statistical significance at a 5% level (*p* < .05), were determined through ANOVA and Duncan's multiple range tests.

## 3 RESULTS AND DISCUSSION

### 3.1 Characteristics of O/W gelled emulsion

The characteristics of O/W gelled emulsions are given in Table 2. The emulsions demonstrated exceptional stability without phase separation during rigorous evaluations, including thermal stability, centrifuge stability, and creaming stability. This indicates their robustness and resistance to thermal stress, centrifugal forces, and gravitational effects. The pH value of the O/W gelled emulsion was measured at 6.30 ± 0.01. Studies involving similar emulsions formulated with chia flour and various gelling agents reported pH values ranging from 6.08 to 6.76, indicating formulation variations. Other studies investigating O/W emulsions with chia oil reported pH values of 6.3 and 6.5, while gelled emulsions containing chia oil and flour showed a pH value of 6.99 ± 0.02 (Herrero et al., 2018; Julio et al., 2015; Pintado et al., 2015). The disparities in pH values can be explained by different gelling techniques and additive variations in the formulations. Regarding color parameters, the beef patties exhibited an L* value of 55.18 ± 0.88, an a* value of 2.24 ± 0.10, and a b* value of 12.78 ± 0.01. In comparison, a study on a gelled emulsion formulation containing chia mucilage, MTG, and carrageenan reported values of 61.92, 4.60, and 22.04 for L*, a*, and b*, respectively (Câmara et al., 2020). The hardness value of the beef patties was determined to be 0.50 ± 0.18 N, while the gumminess value was calculated as 0.21 ± 0.08 N.

**TABLE 2:** Characteristics of O/W gelled emulsion.

Thermal stability	No phase separation
Centrifuge stability	
pH	6.30 ± 0.01
L*	55.18 ± 0.88
a*	2.24 ± 0.10
b*	12.78 ± 0.01
Hardness (N)	0.50 ± 0.18
Gumminess (N)	0.21 ± 0.08

### 3.2 Chemical composition and energy calculations

Table 3 presents a comprehensive analysis of the chemical composition and energy values of various beef patty treatments. Significant differences (*p* < .05) were notably observed in the chemical composition across all treatment groups, except for the ash content. As the fat replacement level increased, there was a discernible rise (*p <* .05) in both moisture and crude fiber contents, while protein and fat contents exhibited a corresponding decrease (*p <* .05). A noteworthy technique for enhancing the nutritional profile by reducing fat content is the structuring of edible oils using gelled emulsions (Öztürk‐Kerimoğlu et al., 2021). Consequently, the reduced fat content observed in our reformulated treatments can be explained by the incorporation of such gelled emulsions, wherein the oil phase constituted only 52% of the emulsion's composition. Of the treatment groups, the G100 displayed the highest moisture and crude fiber contents, alongside the lowest fat content (*p <* .05). The observed increase in moisture content in the reformulated patties could plausibly be ascribed to the inclusion of gelled emulsions during formulation, resulting in an additional influx of water. Similar findings were also documented by Badar et al. (2023) in their study investigating patties formulated with gelled emulsions incorporating chia flour, walnut, or peanut oils. Correspondingly, our previous studies on the use of GE in different meat products had similar outcomes (Kavuşan et al., 2020; Nacak et al., 2021; Serdaroğlu et al., 2017).

**TABLE 3:** Chemical composition of beef patties formulated with an O/W gelled emulsion.

Parameters	Treatments				SEM	*p*‐Value
	C	G50	G75	G100		
Moisture (%)	59.46^d^	62.90^c^	64.36^b^	66.80^a^	0.82	.003
Fat (%)	18.33^a^	16.18^b^	15.06^c^	12.94^d^	0.60	<.001
Protein (%)	19.67^a^	18.70^b^	14.76^c^	14.43^c^	0.71	<.001
Ash (%)	2.94	2.92	3.00	3.07	0.02	.880
Crude fiber (%)	2.16^d^	2.78^c^	3.10^b^	3.29^a^	0.13	<.001
Energy (kcal/g)	255.44^a^	232.09^b^	206.47^c^	186.29^d^	7.96	<.001

*Note*: C: patties prepared with 100% beef fat; G50: patties prepared with 50% gelled emulsion; G75: patties prepared with 75% gelled emulsion; G100: patties prepared with 100% gelled emulsion. ^a–d^Mean values with different superscript letters in a row are statistically different, and values are presented as the mean ± standard error (SEM).

Regarding the energy values, the samples exhibited a range from 186.29 (G100) to 255.44 (C) kcal/g. A substantial reduction in energy content was evident as fat levels diminished (Table 3). Notably, patties containing 100% beef fat exhibited the highest energy value, while samples formulated without beef fat (100% gelled emulsion) demonstrated the lowest value. The G75 and G100 groups achieved notable reductions of over 19% and 27% in energy value, respectively, when compared to the C. A study examining emulsified sausage samples similarly found the highest energy value (249.15 kcal/100 g) in the control group (100% beef fat), while the lowest value (181.57 kcal/100 g) was observed in the group containing 50% gelled emulsion (Nacak et al., 2021).

### 3.3 pH and batter stability

**FIGURE 1:**
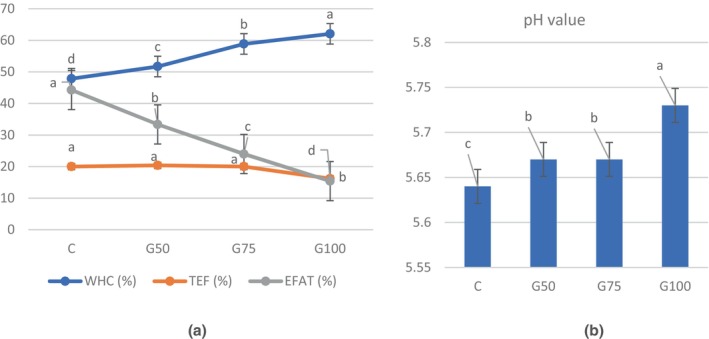
pH and batter stability of patties formulated with an O/W gelled emulsion. C: Patties prepared with 100% beef fat; G50: Patties prepared with 50% gelled emulsion; G75: Patties prepared with 75% gelled emulsion; G100: Patties prepared with 100% gelled emulsion. The values are expressed as means ± SEM. Different letters (a–d) indicate significant differences among groups (*p* < .05). EFAT, Expressible fat; TEF, Total expressible fluid; WHC, Water‐holding capacity.

The pH value of meat patties is illustrated in Figure 1a. The inclusion of GE as a substitute for beef fat exerted a notable impact on the pH value of the beef patties (*p <* .05). It was observed that the pH value of the reformulated patties was higher compared to the control group (C). This increase in pH value can be explained by the higher pH value of the gelled emulsion (6.30), which surpassed the pH of the meat system. A similar trend was reported in patties where pork back fat was completely replaced with chitosan‐stabilized emulsion gels, resulting in higher pH values compared to the control treatment (Cîrstea et al., 2023). The G50 and G75 treatments exhibited similar pH values. The higher pH values observed in the reformulated treatments were associated with an enhanced WHC (Figure 1b). The retention of moisture in the meat, commonly known as WHC, represents a critical parameter in assessing the stability of emulsions.

It assesses the meat's capacity to retain moisture, a critical factor for preserving product quality and texture. (Yüncü et al., 2022). The water‐holding capacity of the treatments ranged between 47.84% and 62.05%. Increasing amounts of GE correlated with an increase in WHC (*p* < .05), with the highest WHC observed in G100. Researchers also stated that GE is a promising alternative to achieving better WHC in meat products. The main reason for improved WHC could be the excellent moisture retention ability of chia flour (Yüncü et al., 2022). Chia seeds possess gel‐forming properties due to the presence of uronic acid, allowing them to absorb and retain water up to 27 times their weight (Muñoz et al., 2012). Also, pH value was another major effect on WHC, since The WHC reaches its minimum at the isoelectric point of the muscle proteins.

Figure 1a illustrates the emulsion stability in the raw dough, and it was observed that replacing beef fat with GE had a notable impact, particularly on the EFAT values, which ranged from 15.4% to 44.25%. The higher the percentage of fat replacement, the greater the emulsion stability (*p <* .05). A lower exudate liquid was obtained in model meat systems with GEs containing alginate or whey protein (Câmara et al., 2020). However, the TEF values remained unaffected by the replacement of beef fat up to 100%. The enhanced stability can be ascribed to the inclusion of chia flour and EWP in the formulation of the GE. Chia flour possesses oil‐binding properties, as highlighted by Kulczyński et al. (2019), while egg white powder is known for its high protein content and is responsible for emulsification (Chang et al., 2021). Furthermore, it was observed that the sample G100 exhibited the lowest amount of exudated fluid (*p <* .05).

### 3.4 Fatty acid composition and cholesterol content

The table presents the fatty acid profiles of patties in which a portion or the entirety of beef fat was replaced with a GE incorporating peanut oil as the lipid phase. The utilization of gelled emulsion as a fat replacer resulted in an enhancement of the fatty acid composition in the patties (Table 4). Among all the patties, stearic acid (C16:0) and palmitic acid (C18:0) were the main SFAs detected. As the replacement level of beef fat increased, the total content of SFAs gradually decreased. Notably, a reduction of 33.71%, 46.64%, and 72.04% in SFAs was achieved with 50%, 75%, and 100% substitution of beef fat by the GE, respectively. These changes can be directly attributed to the reduction in fat content in the patties. Patties with 75% and 100% substitution (G75 and G100) exhibited a significant decrease in the SFA content compared to the control. Therefore, these samples align with the health claim of being “reduced in SFA” (European Parliament, 2006), making them beneficial for cardiovascular health. Our findings are in line with various meat products formulated with GE containing healthier oils (Botella‐Martinez et al., 2022; Dias et al., 2022). Furthermore, the levels of monounsaturated fatty acids (MUFAs) and polyunsaturated fatty acids (PUFAs), specifically oleic acid and linoleic acid, respectively, increased with the higher levels of GE in the patties (*p <* .001). The inclusion of peanut oil in the GE formulation, which contains approximately 50% MUFAs, 33% PUFAs, and 14% SFAs, contributed to the predominant changes in the fatty acid profile. Peanut oil predominantly contains oleic acid and linoleic acid as its major fatty acids (Akhtar et al., 2014). Oleic acid, in particular, is highly desirable in meat products as it aids in cholesterol reduction (Daley et al., 2010). The cholesterol content of the patties decreased from 115.13 to 97.29 mg/100 g due to the reduction in fat content. In fermented sausages (Glisic et al., 2019) and emulsion sausages (Nacak et al., 2021), the use of GE resulted in a decrease in cholesterol content.

**TABLE 4:** Fatty acid composition (mg/100 g tissue), total cholesterol content (mg/100 g lipid), and nutritional ratios of the patties.

Parameters	Treatments				SEM	*p*‐Value
	C	G50	G75	G100		
Capric (C10:0)	0.21^a^	0.15^b^	0.14^b^	0.02^c^	0.02	<.001
Lauric (C12:0)	18.53^a^	0.21^b^	0.18^b^	0.04^c^	2.40	
Myristic (C14:0)	4.23^a^	3.35^b^	2.75^c^	0.83^d^	0.38	
Pentadecanoic (C15:0)	0.63^a^	0.53^b^	0.42^c^	0.17^d^	0.05	
Palmitic (C16:0)	26.23^a^	23.82^b^	20.33^c^	13.29^d^	1.47	
Heptadecanoic (C17:0)	1.66^a^	1.44^b^	1.14^c^	0.43^d^	0.14	
Stearic (C18:0)	23.31^a^	20.09^b^	14.96^c^	6.14^d^	1.96	
∑SFA	74.81^a^	49.59^b^	39.92^c^	20.92^d^	5.86	
Tetradecanoic (C14:1)	0.14^c^	0.14^c^	0.17^b^	0.21^a^	0.01	
Palmitoleic (C16:1)	2.32^a^	2.08^b^	1.84^c^	1.37^d^	0.11	
Oleic (C18:1)	37.13^d^	39.52^c^	43.31^b^	51.14^a^	1.60	
Eicosenoic (C20:1)	0.15^d^	0.28^c^	0.51^b^	0.93^a^	0.08	
∑MUFA	39.75^d^	42.02^c^	45.84^b^	53.65^a^	1.59	
Linoleic (C18:2 ω‐6)	2.42^d^	6.52^c^	11.18^b^	20.45^a^	2.02	
Linolenic (C18:3 ω‐3)	0.45^d^	1.51^c^	3.56^b^	4.67^a^	0.50	
∑PUFA	2.87^d^	8.03^c^	14.74^b^	25.12^a^	2.51	
Cholesterol	115.13^a^	105.06^b^	103.51^c^	97.29^d^	1.93	
Nutritional ratios						
PUFA/SFA	0.04^d^	0.16^c^	0.37^b^	1.20^a^	0.14	
ω‐6/ω‐3	5.39^a^	4.33^b^	3.14^c^	4.38^b^	0.24	
AI	1.45^a^	0.75^b^	0.52^c^	0.21^d^	0.14	
TI	2.38^a^	1.63^b^	0.96^c^	0.40^d^	0.22	

*Note*: C: patties prepared with 100% beef fat; G50: patties prepared with 50% gelled emulsion; G75: patties prepared with 75% gelled emulsion; G100: patties prepared with 100% gelled emulsion. ^a–d^Mean values with different superscript letters in a row are statistically different, and values are presented as the mean ± standard error (SEM).

Abbreviations: AI, atherogenic index; MUFA, mono‐unsaturated fatty acids; PUFA, polyunsaturated fatty acids; SFA, saturated fatty acids; TI, thrombogenic index.

The modified samples demonstrated a significant effect (*p* < .001) on all nutritional indices. The ∑PUFA/∑SFA ratio increased significantly from 0.04 to 1.20 for the control sample and G100 patties, respectively (*p* < .001). This indicates that all the patties meet the recommendation set forth by the European Parliament (ratio over 0.4) (European Parliament, 2006). Moreover, the ω‐6/ω‐3 values decreased as the level of fat substitute rose due to the high content of PUFAs derived from peanut oil. Similarly, ω‐6/ω‐3 was decreased in cooked beef burgers by the use of chia oil gelled emulsion to replace animal fat (Botella‐Martinez et al., 2022). The addition of GE also led to an improvement in AI and TI, which are potential promoters of platelet aggregation. To ensure the maintenance of human health and prevent coronary heart disease, AI and TI values of 1 and 0.5, respectively, are recommended (Fernandes et al., 2014). As the indexes were enhanced (Table 4), it can be concluded that patties formulated with GE containing chia flour, egg white powder, and peanut oil can be regarded as a source of healthy meat products in the human diet. The AI index decreased from 0.45 to 0.24, and the TI index decreased from 0.48 to 0.10 in samples formulated with GE. Similarly, Lucas‐González et al. (2020) found notable decreases in the AI and TI indexes in 33% of reformulated samples containing chia oil in a chestnut flour emulsion gel. The AI index decreased from 0.51 to 0.25, and the TI index decreased from 0.67 to 0.19.

### 3.5 Cooking characteristics

Factors such as cooking yield, moisture retention, fat retention, diameter reduction, and shrinkage play a crucial role in the meat industry when predicting the behavior of burger‐type meat products during the cooking process. These cooking characteristics are essential for assessing the overall quality and consumer satisfaction of the final cooked products. The utilization of gelled emulsions significantly affected the cooking properties of the beef patties (Table 5). Overall, the reformulation of patties with GE led to an increase in cooking yield, thickness, moisture retention, and fat retention, while shrinkage and reduction in diameter decreased. The highest cooking yield was observed in G100 (82.01%), while the lowest value was observed in C (74.98%) (*p <* .001). This phenomenon can be attributed to the high dietary fiber (18%–30%) and protein (20%–22%) content of chia flour, which enhances water‐holding capacity (Yüncü et al., 2022). The moisture retention values of the samples ranged from 50.64% (C) to 53.06% (G75), with no statistically significant difference among the reformulated groups (*p >* .05). The fat retention values increased with an increase in the GE ratio in the formulation (*p <* .001). Similar findings were observed in studies reported by Serdaroğlu et al. (2017) and Lucas‐González et al. (2020). An increment in the gelled emulsion ratio in the formulation resulted in a significant decrease in the rate of diameter reduction and an increase in the desired thickness change during cooking (*p <* .001). The swelling of chia flour and EWP in the meat protein matrix led to an increase in the thickness of the patties during cooking. The G100 shows the highest increase in thickness and the lowest reduction in diameter values (*p <* .001). The addition of chia flour to reduced‐fat burger samples has been observed to result in a lower reduction in diameter compared to the control group. This can be explained by the superior moisture and fat retention properties exhibited by chia flour, as well as its ability to improve emulsion stability, thereby preventing shrinkage of the patty samples. During the cooking process, denaturation of meat proteins occurs, resulting in the release of water and fat. This phenomenon contributes to the shrinkage of patties. Utilization of GE decreased shrinkage, and the highest value was found in G100 (*p <* .001). The decrease in fat levels in the samples has resulted in a reduction in shrinkage. Similarly, it has been reported that the use of chia mucilage in meatball samples results in decreased shrinkage values (Yüncü et al., 2021).

**TABLE 5:** Cooking characteristics of beef patties.

Parameters	Treatments				SEM	*p*‐Value
	C	G50	G75	G100		
Cooking yield (%)	74.98^c^	77.84^b^	79.02^b^	82.01^a^	0.82	.001
Moisture retention (%)	50.64^b^	52.58^a^	53.06^a^	52.90^a^	0.36	.028
Fat retention (%)	34.68^d^	50.37^c^	67.70^b^	75.54^a^	4.78	<.001
Reduction in diameter (%)	17.60^a^	10.72^c^	13.57^b^	7.64^d^	1.12	<.001
Change in thickness (%)	6.84^d^	20.88^c^	26.22^b^	28.22^a^	2.53	<.001
Shrinkage (%)	13.93^a^	7.27^b^	7.96^b^	3.73^c^	1.13	<.001

*Note*: C: patties prepared with 100% beef fat; G50: patties prepared with 50% gelled emulsion; G75: patties prepared with 75% gelled emulsion; G100: patties prepared with 100% gelled emulsion. ^a–d^Mean values with different superscript letters in a row are statistically different, and values are presented as the mean ± standard error (SEM).

### 3.6 Texture profile analysis

The textural parameters of the patties are given in Table 6. Utilization of GE has been found to have a significant effect on all textural parameters (*p <* .001). The hardness values ranged from 8.31 to 22.12 N, and the addition of GE resulted in a decrease in the hardness values of the samples (*p <* .001). Samples containing gelled emulsion had lower hardness values, according to other studies (Barros et al., 2021; Cerrón‐Mercado et al., 2022; Serdaroğlu et al., 2017). Similar to hardness values, the gumminess and chewiness values have also been found to be lower in reformulated samples. This observation can be attributed to the lower hardness (0.50 N) and gumminess (0.21 N) values associated with gelled emulsions. When GE is used, there is a decrease in the fat and protein levels of the samples, accompanied by an increase in moisture content. This leads to the production of tender products, which in turn results in lower hardness values. On the other hand, there was no notable difference in hardness values between the G50 and G75 groups, while no significant difference was found in gumminess and chewiness values between the G75 and G100 (*p >* .05). Except for G50, higher springiness values were recorded in treatments (*p <* .001). Some studies have reported an increase in the springiness values of meat products formulated with gelled emulsion (Barros et al., 2021; Pintado et al., 2016). Fluctuations have been observed in the cohesiveness values of the samples. While the highest value was observed in G100, the lowest value was found in G50 (*p <* .001). Regardless of the usage ratios, the incorporation of chia oil and/or hemp oil in gelled emulsions used as fat replacers in beef burgers resulted in an increase in cohesiveness values (Botella‐Martinez et al., 2022). It has been determined that oils added to the product in the form of GE are better dispersed within the emulsion matrices than animal fats, thereby providing the desired texture due to their interaction with proteins.

**TABLE 6:** Textural properties of beef patties.

Parameters	Treatments				SEM	*p*‐Value
	C	G50	G75	G100		
Hardness (N)	22.12^a^	10.72^b^	10.62^b^	8.31^c^	1.26	<.001
Springiness	0.32^c^	0.26^d^	0.37^b^	0.45^a^	0.02	
Cohesiveness	0.35^b^	0.25^c^	0.36^b^	0.40^a^	0.01	
Gumminess (N)	8.03^a^	2.60^c^	3.56^b^	3.34^b^	0.49	
Chewiness (N·mm)	2.85^a^	0.83^c^	1.34^b^	1.30^b^	0.16	

*Note*: C: patties prepared with 100% beef fat; G50: patties prepared with 50% gelled emulsion; G75: patties prepared with 75% gelled emulsion; G100: patties prepared with 100% gelled emulsion. ^a–d^Mean values with different superscripts in a row are statistically different, and values are presented as the mean ± standard error (SEM).

### 3.7 Microstructure

**FIGURE 2:**
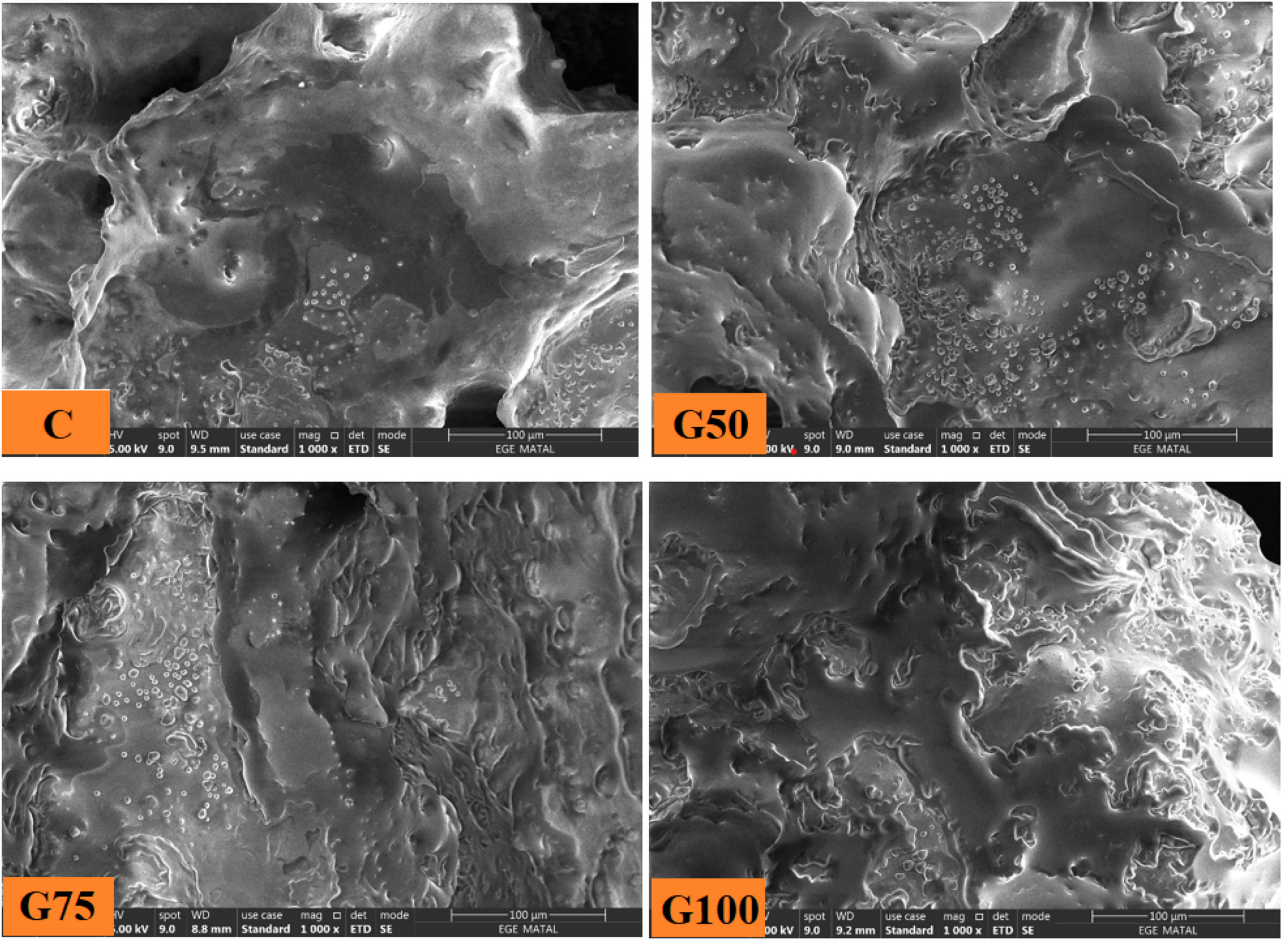
Microstructure of beef patties. C: Patties prepared with 100% beef fat; G50: Patties prepared with 50% gelled emulsion; G75: Patties prepared with 75% gelled emulsion; G100: Patties prepared with 100% gelled emulsion. The size of the particle is 100 μm.

Microstructural images of the patty samples are presented in Figure 2. It is evident that there are voids in the samples of the control group. However, the incorporation of GE has effectively reduced the voids in the structure of beef patties. This observation provides evidence that the inclusion of gelled emulsion in beef patties enhances cooking yield, resulting in a more compact structure. The G100 group, formulated with 100% GE, exhibited a visually homogeneous appearance, suggesting superior distribution within the sample compared to animal fat. Consistent with our findings, burger samples formulated with oleogel containing sesame oil as a replacement for beef fat showed a more compact appearance compared to the control (Moghtadaei et al., 2018). The G50 and G75 groups, characterized by the combination of beef fat and gelled emulsion, exhibited comparable visual characteristics, indicating a resemblance in their overall appearances.

### 3.8 Sensory assessment

Sensory properties can be a limiting factor for fat reduction strategies in meat products due to the various functions fat provides in these products. The results of the sensory assessment are shown in Table 7. No noteworthy differences were noted in the scores of appearance and color among the treatments. (*p >* .05). Similar findings have been reported in a study where amaranth flour was added along with chia oil and/or hemp oil in gelled emulsions to beef burgers (Botella‐Martinez et al., 2022). Reformulated treatments showed higher (*p <* .01) scores for texture, juiciness, and flavor. This observation is consistent with the results of the texture profile analysis (Table 6). G75 and G100 were more acceptable by panelists in terms of oiliness and overall acceptability (*p <* .05). It has been observed that the addition of GE improved the oiliness and overall acceptability values regardless of the usage amount (*p <* .05). When considering all parameters, the replacement of beef fat with GE containing chia flour and peanut oil did not have a negative impact on the sensory parameters of the beef patties. Furthermore, replacing 75% or more of animal fat with GE was preferred by consumers. Contrary to our findings, previous research studies have reported negative effects on sensory characteristics when 100% of animal fat is replaced with gelled emulsion (Pintado et al., 2015, 2016; Serdaroğlu et al., 2017). This difference in findings could be ascribed to variations in the GE formulations and sources of meat used by different researchers in their studies.

**TABLE 7:** Sensory scores of beef patties.

Parameters	Treatments				SEM	*p*‐Value
	C	G50	G75	G100		
Appearance	8.10	8.00	8.50	8.70	0.12	.129
Color	8.10	7.90	8.30	8.50	0.13	.373
Texture	6.20^b^	7.40^a^	7.90^a^	8.30^a^	0.23	.003
Juiciness	6.20^b^	7.30^a^	7.90^a^	8.30^a^	0.22	.002
Oiliness	6.70^b^	7.70^ab^	7.80^ab^	8.10^a^	0.23	.150
Flavor	6.60^b^	7.60^ab^	8.00^a^	8.50^a^	0.22	.010
Overall acceptance	7.10^b^	7.60^ab^	7.90^a^	8.40^a^	0.16	.025

*Note*: C: patties prepared with 100% beef fat; G50: patties prepared with 50% gelled emulsion; G75: patties prepared with 75% gelled emulsion; G100: patties prepared with 100% gelled emulsion. ^a–c^Mean values with different superscripts in a row are statistically different (*p* < .05); ^X–T^Mean values with different superscript letters in a column are statistically different, and values are presented as the mean ± standard error (SEM).

### 3.9 Color

A comprehensive comparative study was carried out to evaluate the color parameters of products with GE and control treatments. Significant changes were observed in the L*, a*, and b* values (Table 8), indicating that substituting beef fat with GE ingredients can yield a color profile similar to the control. Initially, the L* values ranged from 35.8 to 37.4, with no significant differences among C, G50, and G75 (*p* < .05); meanwhile, G100 exhibited the lowest L* value. Incorporating GE with maca flour, soybean oil, and essential oil increased L* values in pork backfat‐patty substitutes (Cerrón‐Mercado et al., 2022). Chia flour, egg white powder, and peanut oil may contribute to a darker appearance due to their color properties. Over storage, L* values fluctuated but remained higher compared to initial values, except for G100, which remained stable over 4 months. No significant color changes were observed in the final storage month, indicating color stability. Increased L* values during storage can be attributed to oxidation and enzymatic reactions, resulting in a lighter appearance.

**TABLE 8:** Color parameters of beef patties.

Parameters	Storage (month)	Treatments				SEM	*p*‐Value
		C	G50	G75	G100		
L*	0	36.7^a,T^	37.3^a,Z^	37.4^a,Y^	35.8^b^	0.20	.003
	1	49.5^a,Y^	39.7^b,Y^	38.6^b,Y^	35.5^c^	1.41	<.001
	2	44.1^a,Z^	40.1^b,Y^	39.0^b,Y^	37.9^b^	0.70	.004
	3	54.7^a,X^	42.1^b,X^	38.8^c,Y^	36.6^c^	1.86	<.001
	4	42.8^Z^	37.8^Z^	41.5^X^	38.1	0.68	.005
	*SEM*	*1*.*49*	*0*.*46*	*0*.*37*	*0*.*37*		
	*p‐value*	*<*.*001*	*<*.*001*	.*001*	.*063*		
a*	0	12.8^a,X^	11.8^b,X^	12.2^b,X^	10.8^c,X^	0.20	<.001
	1	5.5^b,Y^	6.2^a,Y^	6.5^a,Y^	7.0^a,Y^	0.18	.325
	2	5.8^Y^	5.7^Y^	5.8^Z^	6.1^Z^	0.15	.912
	3	3.6^b,Z^	6.1^a,Y^	5.8^a,Z^	6.0^a,Z^	0.28	<.001
	4	6.0^Y^	5.9^Y^	4.6^T^	5.3^T^	0.24	.098
	*SEM*	*0*.*74*	*0*.*55*	*0*.*63*	*0*.*45*		
	*p‐value*	*<*.*001*	*<*.*001*	*<*.*001*	*<*.*001*		
b*	0	8.2^d,XY^	10.0^c^	11.7^a,X^	10.8^b,X^	0.35	<.001
	1	7.6^b,Y^	10.6^a^	10.0^a,Y^	10.2^a,XY^	0.23	<.001
	2	7.1^b,Y^	9.2^a^	8.6^ab,ZT^	10.1^a,XY^	0.41	.035
	3	9.5^X^	10.2	9.5^YZ^	8.9^YZ^	0.24	.365
	4	7.2^Y^	9.2	7.6^T^	8.5^Z^	0.28	.028
	*SEM*	*0*.*28*	*0*.*21*	*0*.*35*	*0*.*26*		
	*p‐value*	.*02*	.*058*	*<*.*001*	.*003*		

*Note*: C: patties prepared with 100% beef fat; G50: patties prepared with 50% gelled emulsion; G75: patties prepared with 75% gelled emulsion; G100: patties prepared with 100% gelled emulsion. ^a–d^Mean values with different superscript letters in a row are statistically different, and values are presented as the mean ± standard error (SEM). The parts written in italics represent the *SEM* and *p‐values* for L*, a*, and b* values of each group throughout storage. The SEM and p‐values not written in italics indicate the significance of the differences between the groups.

The initial a* values ranged from 10.8 to 12.8 (Table 8), with a decrease observed when using GE ingredients. G100 had the lowest initial a* value. During storage, all samples experienced a significant decline in a* values in the first month. The a* values of G75 and G100 showed a decreasing trend over time. However, no significant differences were found between treatments at the end of storage, indicating color convergence. The initial b* values ranged from 8.2 to 11.7, and GE inclusion increased these values regardless of the GE level. G75 had the highest initial b* value. Storage time affected b* values, with a significant decline in all samples in the first month. G75 and G100 showed further decreases over time. Substituting pork backfat with hydrogelled emulsion from linseed oil and pea protein increased b* values (de Lima Guterres et al., 2023). Similar to L* and a* values, no significant differences were found between samples during the 3rd and 4th months of storage, indicating color stability. The changes in a* and b* values with the addition of GE containing chia flour, egg white powder, and peanut oil can be attributed to their specific color properties and interactions. Sausages with GE showed increased L* and decreased a* values compared to the control (de Souza Paglarini et al., 2021). Decreases in a* and increases in b* values during storage may be influenced by oxidation, pigment degradation, enzymatic reactions, or changes in color component structure.

### 3.10 Lipid oxidation

**FIGURE 3:**
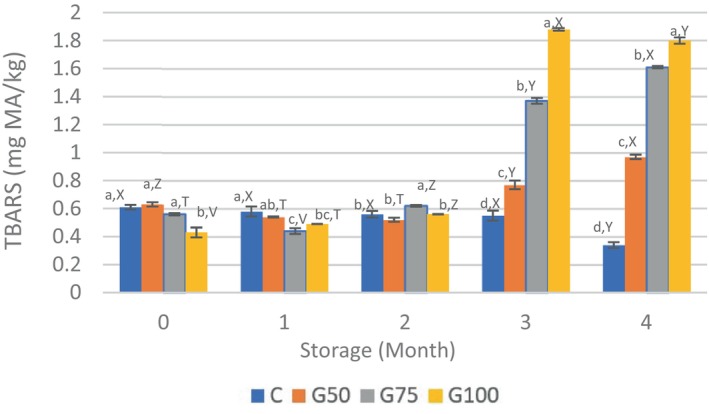
TBARS (mg MA/kg) values of beef patties. ^a–d^Mean values with different superscript letters in a row are statistically different (*p* < .05). ^X–V^Mean values with different superscript letters in a column are statistically different, and values are presented as the mean ± standard error (SEM).

Lipid oxidation affects the quality and shelf life of meat products (Domínguez et al., 2019). TBARS values, an indicator of lipid peroxidation, ranged from 0.34 (C) to 1.88 (G100) mg MA/kg (Figure 3). The control and lower substitution rate GE samples showed no significant changes (*p* > .05); conversely, the GE sample with the highest substitution rate exhibited the lowest TBARS values at the initiation of the storage period (*p* < .01). On the other hand, samples formulated with GE in the 3rd and 4th months of storage were found to have higher TBARS values compared to the C (*p* < .05). It is believed that this is due to the high content of PUFAs in peanut oil, resulting in a higher rate of lipid oxidation. Nevertheless, during the storage period, none of the sample groups exceeded the prescribed limit for TBARS (<2 mg MA/kg, Witte et al., 1970), indicating that lipid oxidation remained within acceptable levels. Despite its high unsaturated fatty acid content, the non‐exceedance of the limit value for lipid oxidation can be attributed to the high presence of phenolic compounds in chia flour. Similar to our results, studies on agar emulsion gels replacing pork fat showed increased TBARS values in Frankfurter sausages, but all samples remained below the defined limit (Fontes‐Candia et al., 2023). Therefore, replacing beef fat with GE appears to be favorable for maintaining the oxidative quality of frozen, stored beef patties.

### 3.11 DSC measurements

The implementation of Differential Scanning Calorimetry (DSC) analysis enabled the acquisition of a comprehensive understanding of the lipid oxidation process in beef patties. The parameters used to evaluate the resistance of the tested samples to thermal oxidative decomposition were the induction time and onset oxidation temperature (OOT) values. In the DSC curves, the initial process at *T*_0_ corresponds to the OOT, while the second process, represented by the peak temperature, defines the thermal decomposition of fats (Ostojić et al., 2016).

**FIGURE 4:**
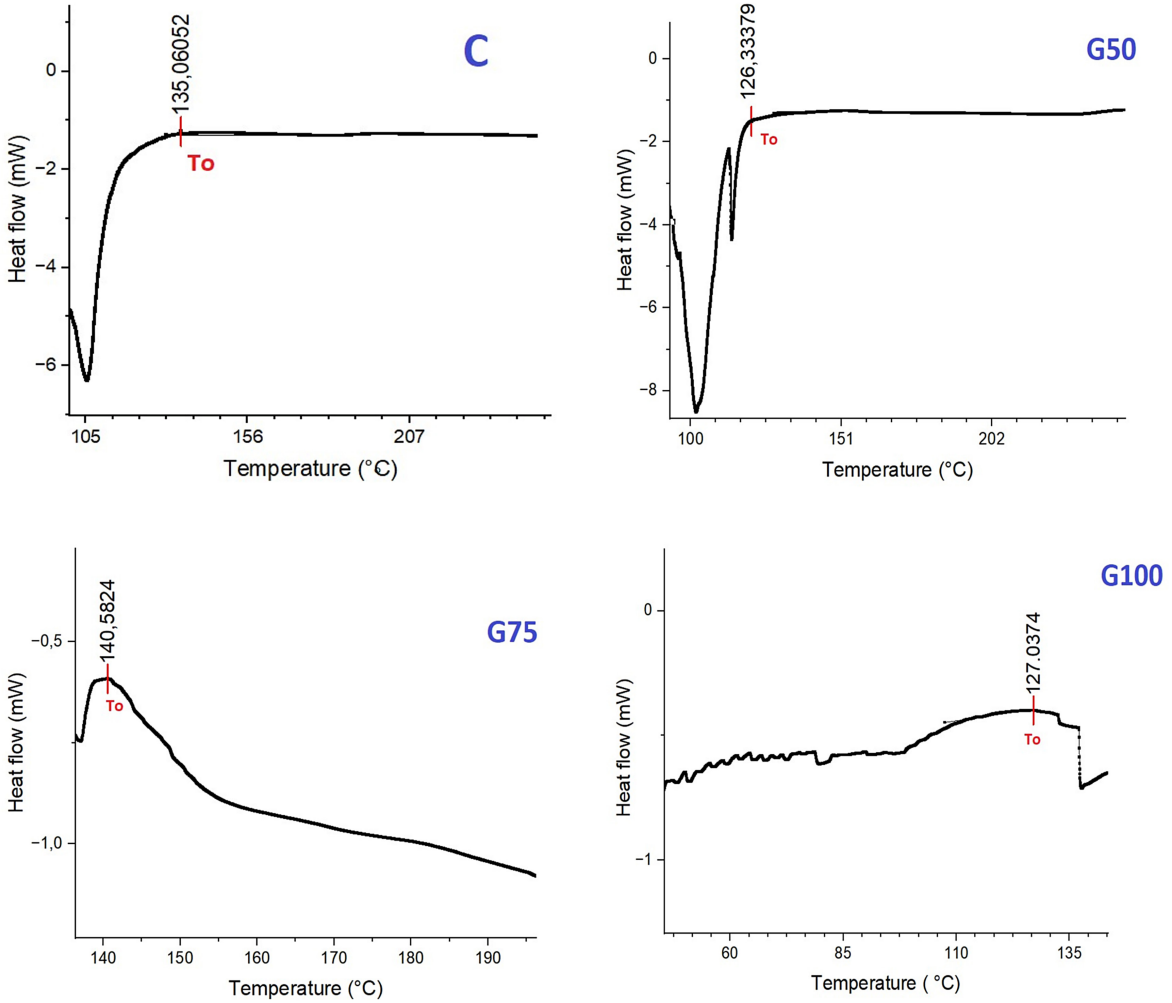
DSC thermograms of beef patties (oxidation onset temperature shown by red arrows as *T*_0_). C: Patties prepared with 100% beef fat; G50: Patties prepared with 50% gelled emulsion; G75: Patties prepared with 75% gelled emulsion; G100: Patties prepared with 100% gelled emulsion.

Figure 4 illustrates the DSC thermograms of the treatments, displaying their respective OOTs. The control treatments, formulated exclusively with beef fat, exhibited an OOT of 135.06°C. Conversely, the reformulated samples containing 50% GE, 75% GE, and 100% GE displayed average OOT values of 126.33, 140.58, and 127.04°C, respectively. In this case, both G75 and C demonstrated higher OOT values compared to the other samples. On the other hand, the OOT values of the G50 and G100 samples were similar. Apart from G75, the elevated OOT values, when compared to the control, can be explained by the increased content of unsaturated fatty acids, which accelerate the oxidation process. The substitution of 75% of beef fat with gel emulsion led to improved retention of fat particles within the meat matrix, consequently offering protection against oxidation (Wirkowska‐Wojdyła et al., 2019). Similarly, in a model system of meat emulsions, it was observed that incorporating beef fat with a pea protein–agar‐agar gel complex at various ratios resulted in higher OOT values compared to the control group (Öztürk‐Kerimoğlu, 2021).

## 4 CONCLUSION

The objective of this study was to investigate how emulsion gel can imitate beef animal fat when used as a substitute in patty formulation. The study's findings showed that the utilization of gelled emulsion as a fat replacer improved the technological and sensory properties of patties. The inclusion of gelled emulsion led to a decrease in fat content, consequently enhancing the nutritional profile. This leads to decreased energy values and enhanced fatty acid composition. Despite higher oxidation levels in final products containing GE, the reformulation process had the potential to offset oxidative stability changes over extended storage periods. These findings suggest that utilizing gelled emulsion prepared with peanut oil, chia flour, and egg white powder holds great potential as an ingredient for creating healthier meat products. Further studies investigating the effects of gelled emulsions containing varying ratios of other vegetable oils on the physicochemical, nutritional, and sensory properties of other meat products such as frankfurters, fermented sausages, and nuggets would be beneficial.

## AUTHOR CONTRIBUTIONS

**Meltem Serdaroğlu:** Conceptualization (equal); investigation (equal); methodology (lead); resources (equal); supervision (lead); visualization (equal); writing – original draft (equal); writing – review and editing (equal). **Özlem Yüncü‐Boyacı:** Conceptualization (equal); formal analysis (lead); investigation (equal); project administration (lead); resources (equal); visualization (equal); writing – original draft (equal); writing – review and editing (equal). **Hülya Serpil Kavuşan:** Formal analysis (equal); investigation (equal); resources (equal); writing – original draft (equal); writing – review and editing (equal).

## CONFLICT OF INTEREST STATEMENT

The authors declare no conflict of interest for this article.

## Data Availability

All data can be obtained from the corresponding author upon reasonable request.
